# The Role of Social Determinants of Health in Self-Reported Psychological Distress among United States Adults Post-COVID-19 Pandemic

**DOI:** 10.3390/ijerph21091219

**Published:** 2024-09-17

**Authors:** Kingsley Kalu, Gulzar H. Shah, Elizabeth Ayangunna, Bushra Shah, Nandi Marshall

**Affiliations:** Department of Health Policy and Community Health, Jiann-Ping Hsu College of Public Health, Georgia Southern University, Statesboro, GA 30458, USA; kk13870@georgiasouthern.edu (K.K.); ea06435@georgiasouthern.edu (E.A.); bs06779@georgiasouthern.edu (B.S.); nmarshall@georgiasouthern.edu (N.M.)

**Keywords:** psychological distress, social determinants of health, COVID-19 post-pandemic, anxiety, depression

## Abstract

Psychological distress, an emotional condition with symptoms of anxiety and depression, leads to impaired function, behavior, and personal traits. The current study examined the association between social determinants of health and the severity of psychological distress among adults in the United States after the COVID-19 pandemic. Using multinomial multivariable logistic regression, we analyzed data from 5106 (*n* = 5106) participants in the Health Information National Trends Survey (HINTS) 6. Compared to non-Hispanic Whites, African Americans (AOR = 0.62, CI = 0.42–0.93) had lower odds of reporting mild psychological distress rather than no stress. Other variables associated with a higher likelihood of reporting moderate to severe psychological distress, rather than no distress, are being in the 50–64 years age group (AOR = 2.77, CI = 1.45–5.28), divorced (AOR = 2.50, CI = 1.70–3.69), and widowed (AOR = 3.78, CI = 2.15–6.64). Respondents living in an urban area had lower odds of reporting moderate to severe psychological distress (AOR = 0.56, CI = 0.39–0.80) compared to those living in rural areas. Our findings identify several risk factors for psychological distress by sociodemographic characteristics such as age, race, marital status, and urban living, providing empirical evidence for interventions in behavioral health. These findings suggest there is an utmost need for a multi-sectoral approach to address the social determinants of health associated with psychological distress post-COVID-19 pandemic.

## 1. Introduction

Psychological distress is a prevalent yet underdiagnosed condition with a potential for negative health consequences [1,2]. A range of depression and anxiety symptoms characterize psychological distress, including low energy levels, sleep disturbance, anger management issues, decreased cognitive functioning, fatigue, memory problems, anhedonia, and sexual dysfunction [2]. Psychological distress has been linked to the development of chronic diseases [3]. According to the *Diagnostic and Statistical Manual of Mental Disorders*, fifth edition (DSM-5), psychological distress is defined as an “undifferentiated group of symptoms ranging from anxiety and depression symptoms to functional impairment, personality traits (confusing, troubling), and behavioral problems” [4]. The symptoms of anxiety (i.e., being restless or tense) and depression (i.e., unhappiness, desperation, and loss of interest) can encompass a diverse range of experiences, from mild symptoms of mental illness to severe psychiatric disease [3,5]. These symptoms may signify the emergence of a somatization disorder, major depressive disorder, suicidal ideation and attempts, academic difficulties, and schizophrenia [6,7].

The increasing prevalence of mental distress in the United States, evidenced by a significant uptick in mental health disorders, highlights a critical public health challenge with widespread implications for individuals and society [8]. The percentage of United States adults reporting severe psychological distress rose from 3.9% in 2018 to 13.6% in 2020 [9]. Studies have also shown an increase in severe psychological distress and outpatient mental health utilization in U.S. adults from 2018 to 2021 [10]. During the COVID-19 pandemic, at least 40% of United States adults encountered elevated levels of psychological distress [11]. Moreover, individuals who experienced depression and anxiety during the COVID-19 pandemic also experienced increased feelings of sadness and hopelessness [12]. In 2023, 50% of adults aged 18–24 in the United States reported depression and anxiety symptoms [12,13]. Also, the healthcare expenditure on mental health services was about USD 280 billion [10,12,14]. Psychological distress can lead to poor health outcomes and the development of several diseases, such as chronic obstructive pulmonary disease, cardiovascular disease, arthritis, suicidal tendencies, and an increased risk of cancer [3,15]. The negative consequences of psychological distress extend beyond individuals to affect families and societies, leading to increased healthcare utilization, reduced quality of life, and potentially higher mortality rates [2,3]. Study shows the loss of a family member due to the COVID-19 pandemic can create psychological distress, leading to conflicts, financial vulnerability, and poor health outcomes [16,17]. Other studies show that the COVID-19 pandemic had a detrimental influence on healthcare workers’ mental well-being with manifestations of stress-related symptoms such as anxiety, depression, sleep disorder, chronic fatigue, and somatic symptoms [18,19].

The social determinants of health (SDoH), encompassing a range of non-medical factors, play a crucial role in shaping health outcomes, particularly when they involve inequities. The SDoH are “the conditions in which people grow, are born, work, live, and the wider set of forces and systems shaping the conditions of daily life”. Social determinants of health can also be the “non-medical factors associated with health outcomes and inequities” [20]. Past studies show that SDoH impacted health inequities during the COVID-19 pandemic [21]. For example, living in an urban community and transportation barriers have been associated with mental health disorders [22,23]. While household income has been linked to lifetime mental disorders and suicide attempts [24], education and marital status are strong predictors of mental health [25,26]. In addition, the absence of health insurance can negatively impact health outcomes by limiting healthcare resources needed to improve health [21,27]. During the COVID-19 pandemic in the United States, a study showed that racial–ethnic disparities in perceived bias and experience of discrimination were associated with psychological distress [28,29]. Another study showed there were disparities in COVID-19-related psychological distress among recipients who utilized state public mental health services [30].

During the COVID-19 pandemic in the United States, several studies examined psychological distress among adults. For instance, Daly and Robinson (2020) examined how psychological distress adapted and whether certain population groups were vulnerable following the COVID-19 pandemic emergence in the United States [31]. Another study assessed the protective role of hope and psychological factors associated with the COVID-19 outbreak [32]. Additionally, some researchers assessed the influence of COVID-19-related stressors, such as occupational difficulties, financial insecurity, and the primary threat of infection in the United States [33].

This study focused on the post-COVID-19 alpha, beta, and delta timeframe due to their influence on viral transmission, mortality, detection, and immune escape, commonly known as the variant of concern [34,35]. In addition, the alpha variants have been associated with higher transmissibility, increased hospitalization, and mortality, while the delta variant was associated with breakthrough infections in vaccinated individuals and caused more severe disease among the unvaccinated [34,36,37]. Given that the COVID-19 pandemic had an impact on mental health, it is essential to assess how SDoH, such as living in the urban community, household income, transportation barriers, marital status, and education are associated with anxiety and depression. The working hypothesis is that social determinants of health are associated with self-reported psychological distress among adults in the United States post-COVID-19 pandemic. This study is unique because it focuses on how the conditions in which people are born or live—the social determinants of health—are related to the severity of anxiety and depression symptoms among adults in the United States after the COVID-19 pandemic. Focusing on the symptoms of anxiety and depression, which will be termed psychological distress, this study aims to assess the association between the SDoH and the severity of psychological distress among United States adults post-COVID-19 pandemic. To better understand the factors associated with psychological distress, this study utilized a validated screening scale to identify the severity of mental health disorders among patients [38,39].

## 2. Materials and Methods

### 2.1. Data Source

This study used secondary data from the Health Information National Trends Survey (HINTS 6). The HINTS is a nationally representative survey routinely administered by the National Cancer Institute (NCI). The HINTS focuses on health issues, including health information, social, telehealth, and genetic testing. Using a cross-sectional study design, the National Cancer Institute conducted this survey to collect nationally representative data among the non-institutionalized United States residents 18 years and older.

### 2.2. Data Collection

HINTS 6 was conducted from 7 March 2022 to 8 November 2022, and data were collected using both paper and online questionnaires in Spanish and English. Selected households received the survey in an initial postal mail and a follow-up reminder, but those that did not respond were sent an additional two reminder mails. To encourage participation, all respondents received a USD 2 pre-paid monetary incentive. The sample size for HINTS 6 was 6252 and only 5106 were included in the final analysis. Respondents with missing data were not included in the analysis. Information regarding sample selection, data collection, and management can be found on the HINTS website: https://hints.cancer.gov/docs/methodologyreports/HINTS_6_MethodologyReport.pdf (accessed on 8 May 2024)

### 2.3. Variables

#### 2.3.1. Dependent Variable

Several validated screening measures for assessing psychological distress include the Kessler-6 distress scale, the Kessler-10 distress scale, and the Distress Questionnaire 5 (D5) based on the *Diagnostic and Statistical Manual of Mental Disorders* (DSM) [40,41]. However, the PHQ-4 screening scale used in the current study is a four-item ultra-brief screening tool that comprises two core criteria for depressive disorder in the Patient Health Questionnaire-2 (PHQ-2) and two core criteria for social anxiety, post-traumatic stress, and panic disorder in the Generalized Anxiety Disorder-7 (GAD-7) questionnaire used in examining the depressive and anxiety disorders [42,43]. Several studies have utilized the PHQ-4 to evaluate the severity of symptoms occurring in depression and anxiety [8,44,45].

The dependent variable, “severity of anxiety and depression”, was based on the screening tool, the Patient Health Questionnaire-4 (PHQ-4). The survey had a stem question, “Over the last two weeks, how often have you been bothered by the following problems?” The problems ranged from anxious or on edge, feeling down, depressed, or hopeless, and having little interest or pleasure in doing things. The PHQ-4 scale consisted of a summation of original categories 0 (not at all), 1 (several days), 2 (more than half the days), or 3 (nearly every day); the total score on PHQ-4 ranged from 0 to 12 [42,43]. For the current study, we used the categorized measure for the PHQ-4 as a guideline [43] and operationalized the PHQ-4 score into three categories—normal or no psychological distress (0–2), mild (3–5), and moderate to severe distress (6–12).

#### 2.3.2. Independent Variables

The independent variables that operationalized the social determinants of health domains are household income (ranging from <USD 20,000 to USD 75,000 or more) to reflect economic stability; health insurance to reflect healthcare access and quality; marital status to capture social and community context; rurality is to capture the respondent’s neighborhood and the built environment. Respondents who identified as living in areas coded as 1–3 in the 2013 USDA rural–urban continuum were categorized as urban residents, and those living in areas coded as 4–8 were categorized as rural residents. Transportation barriers were operationalized by the following survey question: “In the last 12 months, how often did the lack of reliable transportation keep someone in your household from medical appointments, work, or from getting things needed for daily living?” The original categories, often true and sometimes true, were recoded to the presence of transportation barriers, and never true was recoded to the absence of transportation barriers. Educational level is the proxy for education access and quality domain of the SDoH.

Other independent variables of interest are health insurance (yes or no), marital status (married/living with a romantic partner/divorced/widowed/separated/single), education (less than high school/high school graduate/some college/college graduate), household income (less than USD 20,000, USD 20,000 to <USD 35,000, USD 35,000 to <USD 50,000, 50,000 to <USD 75,000, USD 75,000 or more), and rurality (yes/no).

#### 2.3.3. Demographic Variables

The demographic variables of interest are age (18–34 years, 35–49, 50–64, 65–74, and 75 years and older), race (non-Hispanic White/non-Hispanic Black or African American/Hispanic/non-Hispanic Asian/non-Hispanic other), and gender (male/female).

### 2.4. Data Analysis

The weighted percentages and frequencies (unweighted) were computed to describe the survey respondents’ characteristics. Sampling weights were applied to examine the association between PHQ4 score and the social determinants of health. A multinomial multivariable logistic regression analysis was used because the outcome variable had more than two nominal categories. The missing data were handled using listwise deletion of cases with missing values when conducting logistic regression. STATA software version 18 was used in the analysis, and *p* < 0.05 was evaluated as the statistical significance.

## 3. Results

### 3.1. Descriptive Characteristics of the Adult Respondents

Sixty-five percent (65%) of the respondents reported no psychological distress, and twenty-two percent (22%) reported mild psychological distress (Table 1). Forty-six percent (46%) of the study participants were married, whereas 20% were single. A large percentage (87%) lived in an urban area, 49% had a college degree, and 41% earned USD 75,000 or more annually. Additionally, 92% of the respondents had at least one form of health insurance, 89% lacked transportation, 60% were women, and 58% identified their race as non-Hispanic white.

### 3.2. SDoH Associated with Psychological Distress

Results of multinomial multivariable logistic regression (Table 2) show that compared to men, women had higher odds of reporting mild psychological distress (adjusted odds ratio (AOR = 1.43, (confidence interval) CI = 1.13–1.81) and moderate to severe psychological distress (AOR = 1.37, CI = 1.01–1.88), rather than having no distress. Compared to non-Hispanic Whites, non-Hispanic Black/African Americans had lower odds of reporting mild psychological distress (vs. no distress) (AOR = 0.62, CI = 0.42–0.93). Compared to respondents of ages 75 years and above, those in the age groups 18–34 years (AOR = 7.04, CI = 3.34–14.81), 35–49 years (AOR = 5.66, CI = 2.79–11.47), and 50–64 years (AOR = 2.77, CI = 1.45–5.28) had higher odds of reporting moderate to severe psychological distress. However, respondents who were 18–34 years old (AOR = 2.65, CI = 1.52–4.59) and 35–49 years (AOR = 2.03, CI= 1.20–3.45) were more likely to report mild psychological distress (vs. no distress).

Compared to respondents earning less than USD 20,000, those earning USD 75,000 or more had lower odds of reporting moderate to severe psychological distress (AOR = 0.52, CI = 0.283–0.96).

Compared to married respondents, divorced (AOR = 2.50, CI = 1.70–3.69), widowed (AOR = 3.78, CI = 2.15–6.64), and single (AOR = 2.06, CI = 1.35–3.12) respondents had higher odds of reporting moderate to severe psychological distress rather than no psychological distress. Moreover, single respondents (vs. married) were also more likely to report mild psychological distress (AOR = 1.57, CI = 1.13–2.18) rather than no psychological distress. Respondents living in an urban area were less likely to report moderate to severe psychological distress (AOR = 0.56, CI = 0.39–0.80) than respondents living in rural areas. Compared to respondents who did not have transportation barriers, those who had transportation barriers were more likely to report moderate to severe psychological distress (AOR = 5.32, CI = 3.13–9.07) or report mild psychological distress (AOR = 2.82, CI = 1.65–4.84) rather than no psychological distress.

## 4. Discussion

This study provides new findings from the most recent nationally representative survey of U.S. adults using a multivariable multinomial logistic regression. Our results show that transportation barriers, living in urban areas, household income, marital status, and being 18–64 years of age were associated with different levels of psychological distress post-COVID-19 pandemic. Although several studies suggest a link between transportation barriers and depression [46,47], the current study showed that respondents who reported transportation barriers had a five-times higher likelihood of reporting moderate to severe psychological distress and a twofold higher odds of reporting mild psychological distress post-COVID-19 pandemic. The higher odds of distress associated with transportation barriers may be due to a host of factors, such as unaffordable living standards for low-income earners, transportation and infrastructure policies that concentrate on building roads rather than providing means of public transportation, and disproportionate public transit access within the United States [23]. Not having reliable transportation to move oneself or a family member to the hospital for a clinic appointment or to conduct daily living activities such as grocery shopping and going to work can be burdensome and affect the quality of life. Individuals posed with this challenge need to dedicate extra time and finances to organize reliable transportation that could have been easily accessible if they lived in a different neighborhood with higher living standards. There is a need for transportation policies that encourage accessible and reliable public transit systems for all, irrespective of zip code or income level. Transportation systems should also make it easier for caregivers to move their loved ones to clinics, whether in rural or urban settings.

Our study shows that living in an urban area was associated with a decrease in reporting moderate to severe psychological distress post-COVID-19 pandemic. This association might exist because urban area residents have greater access to mental health providers and resources [48]. Also, the end of lockdown and return-to-work policies may have provided a social support system that reduced the psychological distress after the pandemic. However, in contrast, some other studies have also suggested that living in urban areas is linked to an increased risk of mental health disorders such as anxiety and depression [22,48,49]. Urban residents will likely have longer commute hours due to these areas’ industrialization and high population density. Researchers and public health practitioners will need to continue monitoring the severity of psychological distress so tailored interventions can be implemented based on the target population.

The current study also showed that compared to adults 75 years or older, those aged 18–34 years had sevenfold increased odds of reporting moderate to severe psychological distress post-COVID-19 pandemic and had twofold increased odds of reporting mild psychological distress post-COVID-19 pandemic. This pattern of age-related distress might have been because young adults are still experiencing COVID-19 pandemic-related life changes, negative relationships, social isolation, and a lack of counterproductive coping strategies [50,51]. Respondents aged 35–49 years had five times higher odds of reporting moderate to severe psychological distress post-COVID-19 pandemic and twotimes higher odds of reporting mild psychological distress post-COVID-19 pandemic. COVID-19 pandemic-related disruptions to employment and finances may have impacted the mental health of respondents aged 35 to 49 years [9]. The 18–49 age group includes students and the working class, and it is safe to assume the moderate to severe psychological distress reported among this group post-pandemic may be money-related. Securing a job and trying to adjust to workplace post-pandemic policies, e.g., returning to work and possibly having a long commute, may be contributory factors in the reported distress levels among this age group. Respondents 50–64 years were twice as likely to report moderate to severe psychological distress post-COVID-19 pandemic than those aged 75 years or older, potentially attributable to anticipatory anxiety caused by COVID-19 because of the high-risk profile among these age groups [51]. Compared to the 18–49 years age group, the reduced severity of psychological distress among the 50–64 years age group could also be due to reduced financial pressure and being close to retirement. In contrast to the current study, a previous study in the U.S. examined the interaction of age and month of the year, showing that respondents in age groups 18–34 years, 35–49 years, 50–64 years, and 65+ years had reported increased psychological distress in March–April 2020 but claimed their psychological distress returned to pre-pandemic levels by July 2020 [52]. Stakeholders would need to monitor the severity and not just the presence of psychological distress post-pandemic.

Our findings show that identifying as non-Hispanic Blacks was associated with a lower likelihood of post-COVID-19 pandemic psychological distress compared to non-Hispanic Whites. This finding is different from previous studies that have shown that non-Hispanic Blacks have been impacted by mental illness post-COVID-19 pandemic [53,54,55]. The disparity between our study’s results and previous studies may be due to multifactorial factors. Due to the history of discrimination and systemic racism, a large percentage of African Americans may be more likely to have developed race-specific coping strategies to navigate through psychological and socioenvironmental stressors. Examples of these race-coping strategies include being optimistic, religious-based practices, behavior, and interpersonal factors such as religious committees, family, and friends [56,57]. During the COVID-19 pandemic, racial/ethnic minorities were disproportionately impacted by reduced wages and employment, and their household faced economic hardship [58,59], which had a severe effect and overall decline in mental health [12]. The decline in psychological distress post-COVID-19 pandemic may have been due to the likelihood of minority groups relying on federal, state, and other financial support such as the Coronavirus Aid, Relief, and Economic Security (CARES) Act and Paycheck Protection Program, and national initiatives to address COVID-19 disparities among underserved and high-risk communities [60,61,62,63]. There is still a continued need to make health and economic programs easily accessible to everyone, irrespective of their racial identity.

The current study’s results are consistent with past studies that also found a positive association between the influence of relationship status and anxiety, depression, and stress during the COVID-19 pandemic [64]. Previous studies found an increased risk of depression associated with widows [65]; this study also found that widowed respondents had threefold higher odds of reporting moderate to severe psychological distress post-COVID-19 pandemic. This study showed that divorced respondents were twice as likely to report moderate to severe psychological distress post-COVID-19 pandemic compared to married respondents. Moreover, single respondents were two times more likely to report moderate to severe psychological distress and had about twofold higher odds of reporting mild psychological distress post-COVID-19 pandemic. Marriage is a social institution that reduces suicidal risk, substance abuse, anxiety, and depression [66]. The need for companionship and social support is crucial to reducing the levels of psychological distress, and this can be fostered by encouraging relationships at the family, community, and religious levels. Community and online support groups will play a role in ensuring these groups of people can access the social support system needed to reduce psychological distress.

This study conforms to previous studies that female respondents were associated with elevated symptoms of anxiety and depression post-COVID-19 crisis compared to male respondents [12]. Many women have been known to be caregivers of their families, placing a significant burden on them that can contribute to psychological distress [67,68]. This may be due to the increased incidence of gender-based violence and sexual exploitation and decreased maternal and reproductive healthcare services during the COVID-19 pandemic [69]. In addition, increased socioeconomic hardship and reduced social support have been linked to worsening mental health in women [70]. Women should have access to support groups, especially same-sex groups, where they can provide emotional help to each other. Our study also showed that a high household income of about USD 75,000 or more was associated with decreased odds of reporting moderate to severe psychological distress, similar to previous studies [71]. The protective effect of high household income may be because a higher household income has been linked with increased social support, better healthcare, improved employment prospects, and better financial security [72]. Creating an environment where policies enable every member of society to achieve higher standards of living, including higher incomes is necessary for achieving mental health equity.

The authors found no association between health insurance coverage and psychological distress. Previous studies suggest that insured adults with moderate to severe psychological distress had lower odds of receiving mental health treatment despite the availability of insurance coverage [12,73]. Although this study found no association between educational level and psychological distress post-COVID-19 pandemic, past studies suggest that low academic attainment is associated with more significant anxiety–depressive states, perhaps because amid the COVID-19 pandemic, education did not remain a protective factor [33,74,75].

This study’s results should be interpreted given its limitations. We used the PHQ-4 to define the severity of anxiety and depression. While the PHQ-4 is a validated tool for initial screening for distress, certain limitations are associated with it. First, the measurement of anxiety and depression through this tool is not as good as ascertained through clinical diagnosis. In addition, the PHQ-4 may not measure the full spectrum of anxiety and depression symptoms. Thirdly, the social stigma associated with mental health disorders may lead to underreporting of symptoms. In addition, this cross-sectional study cannot support assessing causality between the variables of interest, and a pre- versus post-COVID-19 comparison is not possible. The use of a secondary dataset might limit the variables available for analysis and other psychosocial factors that were not investigated may have influenced the level of psychological distress reported. Regardless of these limitations of our study, its strength is the nationally representative dataset used to examine the factors associated with the severity of mental health distress among adults in the United States post-COVID-19 pandemic.

## 5. Conclusions

Our study showed higher odds of psychological distress for U.S. adults with barriers to transportation, indicating that policymakers need to understand the adverse impacts of transportation barriers manifesting as psychological distress. Acting as the “chief health strategists”, local and state health departments’ leadership should influence policies to ensure everyone has easy access to transportation. To leverage access to transportation as an SDoH, the public health leadership needs to persuade town planners to provide public transit for hard-to-reach areas. Our study findings also suggest that public health professionals should establish screening services for mental health problems and provide community support for those at risk.

An equitable approach to addressing psychological disorders such as distress must involve evaluating other SDoH that may contribute to psychological distress post-COVID-19 pandemic. The local, state, and federal government agencies can leverage the Health-in-All Policies approach to create policies for making mental health professionals more accessible at a low cost. Furthermore, the implementation of stress management behaviors such as nutrition and exercise may help minimize post-COVID-19 psychological distress in the United States. As COVID-19 and other public health emergencies continue to evolve, ongoing surveillance of the levels of psychological distress among the general population and specific populations such as health workers and other essential workers is needed to ensure people can receive the needed help.

## Figures and Tables

**Table 1:** Descriptive statistics of the study respondents’ characteristics, 2022.

Variables	Frequency (*n* = 5106)	% (Weighted)
Dependent variable		
Psychological distress		
Moderate to severe	672	13.2
Mild	1118	21.9
None	3316	64.9
Independent variables		
Marital status		
Married	2351	46.0
Living as married/living with a romantic partner	352	6.9
Divorced	796	15.6
Widowed	491	9.6
Separated	122	2.4
Single, never been married	994	19.5
Urban/rural designation		
Urban	4452	87.2
Rural	654	12.8
Education level		
Less than high school	298	5.8
High school graduate	878	17.2
Some college	1456	28.5
College graduate or more	2474	48.5
Household income		
Less than USD 20,000	816	16.0
USD 20,000 to <USD 35,000	663	13.0
USD 35,000 to <USD 50,000	667	13.1
USD 50,000 to <USD 75,000	880	17.2
USD 75,000 or more	2080	40.7
Health insurance		
Yes	4690	91.8
No	416	8.2
Transportation barriers		
No	587	11.5
Yes	4519	88.5
Age (in years)		
18–34	794	15.6
35–49	1081	21.1
50–64	1501	29.4
65–74	1106	21.7
75+	624	12.2
Gender		
Female	3006	59.7
Male	2032	40.3
Race/ethnicity		
Hispanic	909	17.8
Non-Hispanic White	2938	57.5
Non-Hispanic Black/African American	815	16.0
Non-Hispanic Asian	270	5.3
Non-Hispanic multiple races	174	3.4

**Table 2:** Multinomial multivariable logistic regression analysis of psychological distress measured on PHQ-4 in a sample of 5106 US adults from the 2022 HINTS 6.

	Moderate to Severe Distress (vs. None)				Mild Distress (vs. None)			
		95% Cl				95% Cl		
Independent Variables	AOR	LL	UL	Sig	AOR	LL	UL	Sig
Gender								
Male	(Ref. category)							
Female	**1.37**	1.01	1.88	0.047	**1.43**	1.13	1.81	0.003
Race/Ethnicity								
Non-Hispanic White	(Ref. category)							
Non-Hispanic Black/African American	0.73	0.44	1.22	0.227	**0.62**	0.42	0.93	0.021
Hispanic	0.91	0.51	1.63	0.741	0.73	0.50	1.09	0.121
Non-Hispanic Asian	0.52	0.21	1.29	0.155	1.68	0.89	3.20	0.109
Non-Hispanic other	0.75	0.26	2.18	0.595	0.94	0.47	1.87	0.854
Age								
75 years and older	(Ref. category)							
18–34 years	**7.04**	3.35	14.81	<0.001	**2.65**	1.53	4.59	0.001
35–49 years	**5.66**	2.79	11.47	<0.001	**2.03**	1.20	3.45	0.009
50–64 years	**2.77**	1.45	5.28	0.003	1.37	0.86	2.18	0.183
65–74 years	1.27	0.62	2.60	0.514	1.22	0.86	1.75	0.260
Household Income								
Less than USD 20,000	(Ref. category)							
USD 20,000 to <USD 35,000	0.55	0.29	1.03	0.061	0.88	0.49	1.58	0.661
USD 35,000 to <USD 50,000	0.67	0.37	1.19	0.166	0.92	0.50	1.69	0.788
USD 50,000 to <USD 75,000	0.63	0.36	1.12	0.112	0.78	0.41	1.48	0.443
USD 75,000 or More	**0.52**	0.28	0.96	0.036	0.72	0.38	1.35	0.293
Marital Status								
Married	(Ref. category)							
Living as married/with romantic partner	1.24	0.73	2.10	0.427	1.21	0.79	1.87	0.378
Divorced	**2.50**	1.70	3.69	<0.001	1.30	0.97	1.76	0.081
Widowed	**3.78**	2.16	6.64	<0.001	1.32	0.87	2.01	0.185
Separated	1.75	0.72	4.25	0.212	1.09	0.49	2.42	0.838
Single, never been married	**2.06**	1.35	3.13	0.001	**1.57**	1.13	2.18	0.009
Rurality								
Yes	(Ref. category)							
No	**0.56**	0.39	0.80	0.002	0.95	0.65	1.39	0.775
Transportation Barriers								
No	(Ref. category)							
Yes	**5.33**	3.13	9.07	<0.001	**2.82**	1.65	4.84	<0.001
Health Insurance								
No	(Ref. category)							
Yes	1.44	0.70	2.97	0.318	1.24	0.799	1.924	0.330
Educational Level								
Less than high school	(Ref. category)							
High school graduate	1.56	0.72	3.40	0.256	2.06	0.96	4.43	0.064
Some college	1.39	0.56	3.45	0.476	1.76	0.80	3.84	0.155
College graduate/more	1.37	0.57	3.29	0.474	1.36	0.56	3.30	0.493
The reference outcome category is no psychological distress.								

Note. AOR—adjusted odds ratio; CI—confidence interval; Ref. category—reference category; LL = lower limit; UL = upper limit; Sig—significant level at *p* ≤ 0.05. The boldened number is to highlight significant AOR.

## Data Availability

The dataset is publicly available at https://hints.cancer.gov/ (accessed on 8 May 2024).

## References

[1] Mishra A., Varma A. (2023). A Comprehensive Review of the Generalized Anxiety Disorder. Cureus.

[2] Almansoof A.S., Masuadi E., Al-Muallem A., Agha S. (2024). Prevalence of psychological distress among health sciences students: A systematic review and meta-analysis. Qual. Quant..

[3] McLachlan K.J.J., Gale C.R. (2018). The Effects of Psychological Distress and Its Interaction with Socioeconomic Position on Risk of Developing Four Chronic Diseases. J. Psychosom. Res..

[4] Belay A.S., Guangul M.M., Niguse W., Mesafint G. (2021). Prevalence and Associated Factors of Psychological Distress among Nurses in Public Hospitals, Southwest, Ethiopia: A Cross-Sectional Study. Ethiop. J. Health Sci..

[5] Viertiö S., Kiviruusu O., Piirtola M., Kaprio J., Korhonen T., Marttunen M., Suvisaari J. (2021). Factors Contributing to Psychological Distress in the Working Population, with a Special Reference to Gender Difference. BMC Public Health.

[6] Ashager K., Feleke G.M., Degefu S., Elfios E., Getnet A., Ezo E., Sintayehu M. (2023). Psychological Distress and Associated Factors among Asthmatic Patients in Southern, Ethiopia, 2021. Asthma Res. Pract..

[7] Materu J., Kuringe E., Nyato D., Galishi A., Mwanamsangu A., Katebalila M., Shao A., Changalucha J., Nnko S., Wambura M. (2020). The Psychometric Properties of PHQ-4 Anxiety and Depression Screening Scale among out of School Adolescent Girls and Young Women in Tanzania: A Cross-Sectional Study. BMC Psychiatry.

[8] American Psychological Association Stress in America 2020. https://www.apa.org/news/press/releases/stress/2020/sia-mental-health-crisis.pdf.

[9] McGinty E.E., Presskreischer R., Anderson K.E., Han H., Barry C.L. (2020). Psychological Distress and COVID-19–Related Stressors Reported in a Longitudinal Cohort of US Adults in April and July 2020. JAMA.

[10] Columbia University’s Mailman School of Public Health U.S. Adults Face Distress, Unequal Mental Health Care Access during the COVID-19 Era. News-Medical. https://www.news-medical.net/news/20240206/US-adults-face-distress-unequal-mental-health-care-access-during-the-COVID-19-era.aspx#:~:text=The%20rate%20of%20serious%20psychological.

[11] Pasquini G., Keeter S. At Least Four-in-Ten U.S. Adults Have Faced High Levels of Psychological Distress during COVID-19 Pandemic. Pew Research Center. https://www.pewresearch.org/short-reads/2022/12/12/at-least-four-in-ten-u-s-adults-have-faced-high-levels-of-psychological-distress-during-covid-19-pandemic/.

[12] Panchal N., Saunders H., Rudowitz R., Cox C. The Implications of COVID-19 for Mental Health and Substance Use. KFF. https://www.kff.org/mental-health/issue-brief/the-implications-of-covid-19-for-mental-health-and-substance-use/.

[13] Lee C. Latest Federal Data Show That Young People Are More Likely Than Older Adults to Be Experiencing Symptoms of Anxiety or Depression. KFF. https://www.kff.org/mental-health/press-release/latest-federal-data-show-that-young-people-are-more-likely-than-older-adults-to-be-experiencing-symptoms-of-anxiety-or-depression/#:~:text=The%20analysis%20of%20the%20Census.

[14] The White House Reducing the Economic Burden of Unmet Mental Health Needs. The White House. https://www.whitehouse.gov/cea/written-materials/2022/05/31/reducing-the-economic-burden-of-unmet-mental-health-needs/.

[15] Hockey M., Rocks T., Ruusunen A., Jacka F.N., Huang W., Liao B., Aune D., Wang Y., Nie J., O’Neil A. (2021). Psychological Distress as a Risk Factor for All-Cause, Chronic Disease- and Suicide-Specific Mortality: A Prospective Analysis Using Data from the National Health Interview Survey. Soc. Psychiatry Psychiatr. Epidemiol..

[16] Gayatri M., Puspitasari M.D. (2022). The Impact of COVID-19 Pandemic on Family Well-Being: A Literature Review. Fam. J..

[17] Guttormson J.L., Calkins K., McAndrew N., Fitzgerald J., Losurdo H., Loonsfoot D. (2022). Critical Care Nurse Burnout, Moral Distress, and Mental Health during the COVID-19 Pandemic: A United States Survey. Heart Lung.

[18] Alhouri A., Shokor M.A., Marwa K., Sharabi A., Arrouk D.M.N., Houri F.N.A., Houri H.A., Alhouri A., Shokor M.A., Marwa K. (2023). COVID-19 and Its Impact on Healthcare Workers: Understanding Stigma, Stress, and Quality of Life. Cureus.

[19] Zara G., Settanni M., Zuffranieri M., Veggi S., Castelli L. (2021). The Long Psychological Shadow of COVID-19 upon Healthcare Workers: A Global Concern for Action. J. Affect. Disord..

[20] Centers for Disease Control and Prevention Social Determinants of Health. Centers for Disease Control and Prevention. https://www.cdc.gov/about/priorities/social-determinants-of-health-at-cdc.html?CDC_AAref_Val=https://www.cdc.gov/about/sdoh/index.html.

[21] US Department of Health and Human Services Access to Health Services. Healthy People 2030. https://health.gov/healthypeople/priority-areas/social-determinants-health/literature-summaries/access-health-services.

[22] Srivastava K. (2009). Urbanization and Mental Health. Ind. Psychiatry J..

[23] Mills S., The Link between Mental Health and Transportation Barriers Health Care Access Now. https://healthcareaccessnow.org/the-link-between-mental-health-and-transportation-barriers/.

[24] Sareen J., Afifi T.O., McMillan K.A., Asmundson G.J.G. (2011). Relationship between Household Income and Mental Disorders. Arch. Gen. Psychiatry.

[25] Jace C.E., Makridis C.A. (2021). Does Marriage Protect Mental Health? Evidence from the COVID-19 Pandemic. Soc. Sci. Q..

[26] Williams N. How Does Education Affect Mental Health? News-Medical.net. https://www.news-medical.net/health/How-does-Education-Affect-Mental-Health.aspx#:~:text=Higher%20levels%20of%20education%20have.

[27] Benda N.C., Veinot T.C., Sieck C.J., Ancker J.S. (2020). Broadband Internet Access Is a Social Determinant of Health!. Am. J. Public Health.

[28] Strassle P.D., Wilkerson M.J., Stewart A.L., Forde A.T., Jackson C.L., Singh R., Nápoles A.M. (2023). Impact of COVID-Related Discrimination on Psychological Distress and Sleep Disturbances across Race-Ethnicity. J. Racial Ethn. Health Disparities.

[29] Wen M., Shi L., Zhang D., Li Y., Chen Z., Chen B., Chen L., Zhang L., Li H., Li J. (2023). Racial-Ethnic Disparities in Psychological Distress during the COVID-19 Pandemic in the United States: The Role of Experienced Discrimination and Perceived Racial Bias. BMC Public Health.

[30] Ehntholt A., Rodgers I., Lekas H., Lewis-Fernández R., Samaranayake D., Anderson A., Capobianco L., Cohen D.E., Feeney S., Leckman-Westin E. (2023). Disparities in COVID-19–Related Psychological Distress among Recipients of a State’s Public Mental Health Services. Psychiatr. Serv..

[31] Daly M., Robinson E. (2020). Psychological Distress and Adaptation to the COVID-19 Crisis in the United States. J. Psychiatr. Res..

[32] Flesia L., Adeeb M., Waseem A., Helmy M., Monaro M. (2023). Psychological Distress Related to the COVID-19 Pandemic: The Protective Role of Hope. Eur. J. Investig. Health Psychol. Educ..

[33] Zheng J., Morstead T., Sin N., Klaiber P., Umberson D., Kamble S., DeLongis A. (2021). Psychological Distress in North America during COVID-19: The Role of Pandemic-Related Stressors. Soc. Sci. Med..

[34] Luo C.H., Morris C.P., Sachithanandham J., Amadi A., Gaston D., Li M., Swanson N.J., Schwartz M., Klein E.Y., Pekosz A. (2021). Infection with the Severe Acute Respiratory Syndrome Coronavirus 2 (SARS-CoV-2) Delta Variant Is Associated with Higher Recovery of Infectious Virus Compared to the Alpha Variant in Both Unvaccinated and Vaccinated Individuals. Clin. Infect. Dis..

[35] Salehi-Vaziri M., Fazlalipour M., Seyed Khorrami S.M., Azadmanesh K., Pouriayevali M.H., Jalali T., Shoja Z., Maleki A. (2022). The Ins and Outs of SARS-CoV-2 Variants of Concern (VOCs). Arch. Virol..

[36] Florensa D., Mateo J., Spaimoc R., Miret C., Godoy S., Solsona F., Godoy P. (2022). Severity of COVID-19 Cases in the Months of Predominance of the Alpha and Delta Variants. Sci. Rep..

[37] Tabatabai M., Juarez P.D., Matthews-Juarez P., Wilus D.M., Ramesh A., Alcendor D.J., Tabatabai N., Singh K.P. (2023). An Analysis of COVID-19 Mortality during the Dominancy of Alpha, Delta, and Omicron in the USA. J. Prim. Care Community Health.

[38] Khubchandani J., Brey R., Kotecki J., Kleinfelder J., Anderson J. (2016). The Psychometric Properties of PHQ-4 Depression and Anxiety Screening Scale among College Students. Arch. Psychiatr. Nurs..

[39] Löwe B., Wahl I., Rose M., Spitzer C., Glaesmer H., Wingenfeld K., Schneider A., Brähler E. (2010). A 4-Item Measure of Depression and Anxiety: Validation and Standardization of the Patient Health Questionnaire-4 (PHQ-4) in the General Population. J. Affect. Disord..

[40] Batterham P.J., Sunderland M., Carragher N., Calear A.L., Mackinnon A.J., Slade T. (2016). The Distress Questionnaire-5: Population Screener for Psychological Distress Was More Accurate than the K6/K10. J. Clin. Epidemiol..

[41] Kessler R.C., Green J.G., Gruber M.J., Sampson N.A., Bromet E., Cuitan M., Furukawa T.A., Gureje O., Hinkov H., Hu C.-Y. (2010). Screening for Serious Mental Illness in the General Population with the K6 Screening Scale: Results from the WHO World Mental Health (WMH) Survey Initiative. Int. J. Methods Psychiatr. Res..

[42] Kroenke K., Spitzer R.L., Williams J.B.W., Löwe B. (2009). An Ultra-Brief Screening Scale for Anxiety and Depression: The PHQ–4. Psychosomatics.

[43] Wicke F.S., Krakau L., Löwe B., Beutel M.E., Brähler E. (2022). Update of the Standardization of the Patient Health Questionnaire-4 (PHQ-4) in the General Population. J. Affect. Disord..

[44] Mathur S., Okal J., Musheke M., Pilgrim N., Kishor Patel S., Bhattacharya R., Jani N., Matheka J., Banda L., Mulenga D. (2018). High Rates of Sexual Violence by Both Intimate and Non-Intimate Partners Experienced by Adolescent Girls and Young Women in Kenya and Zambia: Findings around Violence and Other Negative Health Outcomes. PLoS ONE.

[45] Stanhope J. (2016). Patient Health Questionnaire-4. Occup. Med..

[46] Lee L.H., Yoon Y.J., Kim D., Noh H., Jones S., Lee H.Y. (2023). Perceived Transportation Barriers Moderate the Association between Depressive Symptoms and Household Transportation Use: A Pilot Study. J. Transp. Health.

[47] Lyeo J.S., Tiznado-Aitken I., Farber S., Brown H.K., Spence N. (2023). Predictors of Transportation-Related Barriers to Healthcare Access in a North American Suburb. J. Public Health.

[48] Matiullah S., Généreux M., Petit G. (2020). Rural and Urban Variation in Psychological Distress among Adults: Results of the 2014–2015 Eastern Townships Population Health Survey (ETPHS). Can. J. Public Health.

[49] Okubo R., Yoshioka T., Malaya T., Hanibuchi T., Okano H., Ikezawa S., Tsuno K., Murayama H., Tabuchi T. (2021). Urbanization Level and Neighborhood Deprivation, Not COVID-19 Case Numbers by Residence Area, Are Associated with Severe Psychological Distress and New-Onset Suicidal Ideation during the COVID-19 Pandemic. J. Affect. Disord..

[50] Birditt K.S., Turkelson A., Fingerman K.L., Polenick C.A., Oya A. (2020). Age Differences in Stress, Life Changes, and Social Ties during the COVID-19 Pandemic: Implications for Psychological Well-Being. Gerontologist.

[51] Pearman A., Hughes M.L., Smith E.L., Neupert S.D. (2020). Age Differences in Risk and Resilience Factors in COVID-19-Related Stress. J. Gerontol. Ser. B.

[52] Best R., Strough J., Bruine de Bruin W. (2023). Age Differences in Psychological Distress during the COVID-19 Pandemic: March 2020—June 2021. Front. Psychol..

[53] Substance Abuse and Mental Health Service Administration Section 6 PE Tables—Results from the 2021 National Survey on Drug Use and Health: Detailed Tables, SAMHSA, CBHSQ. https://www.samhsa.gov/data/sites/default/files/reports/rpt39441/NSDUHDetailedTabs2021/NSDUHDetailedTabs2021/NSDUHDetTabsSect6pe2021.htm.

[54] Taylor I. (2022). COVID-19 and Mental Health Disparities in the Black American Population. HCA Healthc. J. Med..

[55] Miller-Roenigk B., Jester J.K., Stevens-Watkins D., Francis D.B. (2023). Correlates of Psychological Distress among African American Young Adults during the COVID-19 Pandemic. J. Black Psychol..

[56] Al-Amin N.S., McBryde-Redzovic A., Gutierrez-Kapheim M., Mitchell U.A. (2023). COVID-Related Stressors and Psychological Distress among Chicago Residents: The Moderating Role of Race. J. Racial Ethn. Health Disparities.

[57] Mouzon D.M., Taylor R.J., Nguyen A.W., Chatters L.M. (2016). Serious Psychological Distress among African Americans: Findings from the National Survey of American Life. J. Community Psychol..

[58] Alhomsi A., Quintero S.M., Ponce S., Mendez I., Stewart A.L., Napoles A.M., Strassle P.D. (2023). Racial/Ethnic Disparities in Financial Hardship during the First Year of the Pandemic. Health Equity.

[59] Goldman N., Pebley A.R., Lee K., Andrasfay T., Pratt B. (2021). Racial and Ethnic Differentials in COVID-19-Related Job Exposures by Occupational Standing in the US. PLoS ONE.

[60] Centers for Disease Control and Prevention National Initiative to Address COVID-19 Health Disparities Among Population at High-Risk and Underserved, Including Racial and Ethnic Minority Populations and Rural Communities Funding. https://www.cdc.gov/public-health-gateway/php/funding/covid-19-health-disparities-ot21-2103.html.

[61] Kakani P., Chandra A., Mullainathan S., Obermeyer Z. (2020). Allocation of COVID-19 Relief Funding to Disproportionately Black Counties. JAMA.

[62] The White House The American Rescue Plan and Black Communities. https://www.whitehouse.gov/wp-content/uploads/2021/03/arp-fact-sheet-black-communities.pdf.

[63] U.S. Government Accountability Office COVID-19: HHS Funds Allocated to Support Disproportionately Affected Communities. https://www.gao.gov/products/gao-23-105500.

[64] Nkire N., Nwachukwu I., Shalaby R., Hrabok M., Vuong W., Gusnowski A., Surood S., Greenshaw A.J., Agyapong V.I.O. (2021). COVID-19 Pandemic: Influence of Relationship Status on Stress, Anxiety, and Depression in Canada. Ir. J. Psychol. Med..

[65] Brown S.L., Bulanda J.R., Lee G.R. (2005). The Significance of Nonmarital Cohabitation: Marital Status and Mental Health Benefits among Middle-Aged and Older Adults. J. Gerontol. Ser. B Psychol. Sci. Soc. Sci..

[66] Spiker R.L. (2014). Mental Health and Marital Status. The Wiley Blackwell Encyclopedia of Health, Illness, Behavior, and Society.

[67] Herrero R., Díaz A., Zueco J. (2024). The Burden and Psychological Distress of Family Caregivers of Individuals with Autism Spectrum Disorder: A Gender Approach. J. Clin. Med..

[68] Sharma N., Chakrabarti S., Grover S. (2016). Gender Differences in Caregiving among Family—Caregivers of People with Mental Illnesses. World J. Psychiatry.

[69] U.S Global Leadership Coalition COVID-19 BRIEF: Impact on Women and Girls. USGLC.

[70] Di Blasi M., Albano G., Bassi G., Mancinelli E., Giordano C., Mazzeschi C., Pazzagli C., Salcuni S., Lo Coco G., Gelo O.C.G. (2021). Factors Related to Women’s Psychological Distress during the COVID-19 Pandemic: Evidence from a Two-Wave Longitudinal Study. Int. J. Environ. Res. Public Health.

[71] Shields-Zeeman L., Collin D.F., Batra A., Hamad R. (2021). How Does Income Affect Mental Health and Health Behaviours? A Quasi-Experimental Study of the Earned Income Tax Credit. J. Epidemiol. Community Health.

[72] Liu G., Liu W., Zheng X., Li J. (2023). The Higher the Household Income, the Lower the Possibility of Depression and Anxiety Disorder: Evidence from a Bidirectional Mendelian Randomization Study. Front. Psychiatry.

[73] Jacobs A.W., Hill T.D., Burdette A.M. (2014). Health Insurance Status and Symptoms of Psychological Distress among Low-Income Urban Women. Soc. Ment. Health.

[74] Joannès C., Redmond N.M., Kelly-Irving M., Klinkenberg J., Guillemot C., Sordes F., Delpierre C., Neufcourt L. (2023). The Level of Education Is Associated with an Anxiety-Depressive State among Men and Women—Findings from France during the First Quarter of the COVID-19 Pandemic. BMC Public Health.

[75] Muñoz I.G., Santos-Lozada A.R. (2021). Educational Attainment and Psychological Distress among Working-Age Adults in the United States. SSM Ment. Health.

